# Working Memory-Related Functional Brain Patterns in Never Medicated Children with ADHD

**DOI:** 10.1371/journal.pone.0049392

**Published:** 2012-11-14

**Authors:** Isabelle Massat, Hichem Slama, Martin Kavec, Sylvie Linotte, Alison Mary, Daniele Baleriaux, Thierry Metens, Julien Mendlewicz, Philippe Peigneux

**Affiliations:** 1 UR2NF - Neuropsychology and Functional Neuroimaging Research at CRCN- Center for Research in Cognition and Neurosciences and UNI - ULB Institute for Neurosciences, Université Libre de Bruxelles (ULB), Bruxelles, Belgium; 2 Laboratory of Experimental Neurology, Université Libre de Bruxelles (ULB), Bruxelles, Belgium; 3 INSERM U894 Team 1, Centre de Psychiatrie et de Neurosciences (CPN), Paris, France; 4 Department of Radiology, Clinics of Magnetic Resonance, ULB Erasme Hospital, Bruxelles, Belgium; 5 Department of Child and Adolescent Psychopathology, Robert Debre Hospital, Paris, France; Bellvitge Biomedical Research Institute-IDIBELL, Spain

## Abstract

Attention Deficit/Hyperactivity Disorder (ADHD) is a pervasive neurodevelopmental disorder characterized by 3 clusters of age-inappropriate cardinal symptoms: inattention, hyperactivity and impulsivity. These clinical/behavioural symptoms are assumed to result from disturbances within brain systems supporting executive functions including working memory (WM), which refers to the ability to transiently store and flexibly manipulate task-relevant information. Ongoing or past medications, co-morbidity and differences in task performance are potential, independent confounds in assessing the integrity of cerebral patterns in ADHD. In the present study, we recorded WM-related cerebral activity during a memory updating N-back task using functional Magnetic Resonance Imaging (fMRI) in control children and never medicated, prepubescent children with ADHD but without comorbid symptoms. Despite similar updating performance than controls, children with ADHD exhibited decreased, below baseline WM-related activation levels in a widespread cortico-subcortical network encompassing bilateral occipital and inferior parietal areas, caudate nucleus, cerebellum and functionally connected brainstem nuclei. Distinctive functional connectivity patterns were also found in the ADHD in these regions, with a tighter coupling in the updating than in the control condition with a distributed WM-related cerebral network. Especially, cerebellum showed tighter coupling with activity in an area compatible with the brainstem red nucleus. These results in children with clinical core symptoms of ADHD but without comorbid affections and never treated with medication yield evidence for a core functional neuroanatomical network subtending WM-related processes in ADHD, which may participate to the pathophysiology and expression of clinical symptoms.

## Introduction

Attention Deficit/Hyperactivity Disorder (ADHD) is one of the most common childhood developmental disorder characterized by three clusters of age-inappropriate cardinal symptoms: inattention, hyperactivity and impulsivity. Worldwide prevalence is high (around 5%) [1], and multiple forms are present including the inattentive form, a rare purely hyperactive form, and the most common combined-type that features both inattention and hyperactivity. Although ADHD symptoms have been thought for long to dissipate with puberty, recent studies suggest it a chronic developmental disorder that persists into adulthood in at least 30% of the patients [2]. At the cognitive level, deficits in executive functions, behavioural inhibition and working memory (WM) are key neuropsychological features in the ADHD [3]. WM refers to the ability to transiently store and manipulate information “held online” in the service of complex cognition for further behavioural guidance [4]. It is an outcome of sustained attentional focus on task-relevant mental representations and on suppression of competing distracting events. Effective use of mental representations is critical for behavioural and cognitive flexibility [5] and a sensitive marker of cognitive development [6] strongly associated with academic under achievement [7], [8]. Hence, WM deficits may at least partially subtend clinical symptoms in the ADHD.

The neural patterns associated with WM in adults are well characterized, mostly involving a bilateral parieto-frontal network in the classical N-back task [9]. In healthy children, patterns are both similar [7], [8], [10], [11] and distinctive in showing additional and/or alternate activation patterns in premotor and parietal cortex and insula, and in the striatum and the cerebellum, supposedly reflecting different cognitive strategies and functional brain organization [7]. In adults [12], [13], [14], adolescents [15] and children [16], [17], [18] with ADHD, available studies indicate differential WM-related activation patterns in a distributed set of regions encompassing frontal, parietal and occipital cortices, as well as in the striatum and the cerebellum. These studies shed light on the disorder itself, but also provided new insights onto the mechanisms of normal cognition and attention [19]. However, the identification of core regional cerebral deficits cannot easily account for the substantial heterogeneity observed in ADHD patients with distinctive aetiological profiles [20], and a more promising approach might be the search for deficits in brain pathways possibly leading to the ADHD symptomatology [21]. In this respect, altered patterns of connectivity within the resting state (also called default-mode network [DMN]) [22] have been reported in the ADHD [23], likely related to attentional lapses, WM deficits and task performance variability that are symptomatic of this disorder [24]. To the best of our knowledge, connectivity patterns during WM in children with ADHD compared to healthy children have not been reported so far. Noticeably, high rates of comorbidity [25], ongoing medications and behavioural differences in WM performance are potential confounds in ADHD studies having shown regional decreases in WM-related brain activation, each of these parameters having the potential to impact independently on functions and patterns of cerebral activity involved in WM.

We addressed these issues using fMRI by investigating the cerebral activity subtending WM in prepubescent children presenting or not the clinical symptoms of ADHD, matched for behavioural performance, selected with stringent criteria excluding co-morbidity, and never treated with medication. Results evidence specific functional cerebral patterns subtending WM-related processes in the ADHD, which may underline the pathophysiology and the expression of clinical symptoms.

## Results

Behavioural and neuroimaging analyses were conducted on right-handed children fulfilling the DSM-IV criteria for the ADHD combined type (n = 19) and healthy volunteers (n = 14). Mean age was similar in ADHD (10.75±1.31 years) and Control (10.05±1.28 years) groups (*t = *1.53, *p* = 0.13). All children were scanned using 3T fMRI in a block design during alternating practice between two conditions in the N-back task [9]. In the vigilant/control condition (N0), children pressed a button whenever the number “2” was displayed. In the 2-back, working memory condition (N2), they pressed the button when the displayed number was identical to the number displayed two trials before. Each block consisted of 30 stimuli with 10 target trials. Corrected accuracy scores (hits - false detections/2) were obtained in the N2 and N0 conditions. WM performance reflecting the updating process (UP = N0–N2 corrected scores) was similar between ADHD and control children (UP mean 4.21±2.97 vs. 5.14±2.92; Z = −1.09, p = 0.27), as well as all other performances measures (mean reaction time [RT], RT variability, percentage of correct responses in N2 and N0 conditions; all ps >0.1, see Table 1). Head motion (shifts and rotations) parameters during scanning time were also similar between groups (variability coefficients of translations and rotations, means of rotation and translations; all ps >0.07).

**Table 1:** Behavioral performance.

	Accuracy % (sd)			mean RT (sd)		RT variability	
	N0	N2	N0–N2	N0	N2	N0	N2
ADHD	98.0 (4.6)	92.5 (8.3)	4.21 (.68)	525 (104)	616(155)	.25	.34
CONTROLS	99.6 (1.15)	91.4 (6.0)	5.14 (.78)	552 (66)	698(122)	.25	.37
*p-value (statistic)*	.16 (Z = −1.42)	.29 (Z = −1.06)	.27 (Z = −1.09)	.40 (t = −8.85)	.12 (t = −1.62)	.99 (t = −0.01)	.26 (t = −1.14)

Note. sd = standard deviation of the mean; RT = reaction time; N0 = control identification condition; N2 = N-back 2 condition; RT variability N0 = mean sd in N0/mean RT; RT variability N2 = mean sd in N2/mean RT; Z = Mann-Whitney Test value; t = Student t-test value.

### Decreased Working Memory-related Activity in ADHD

**Figure 1:**
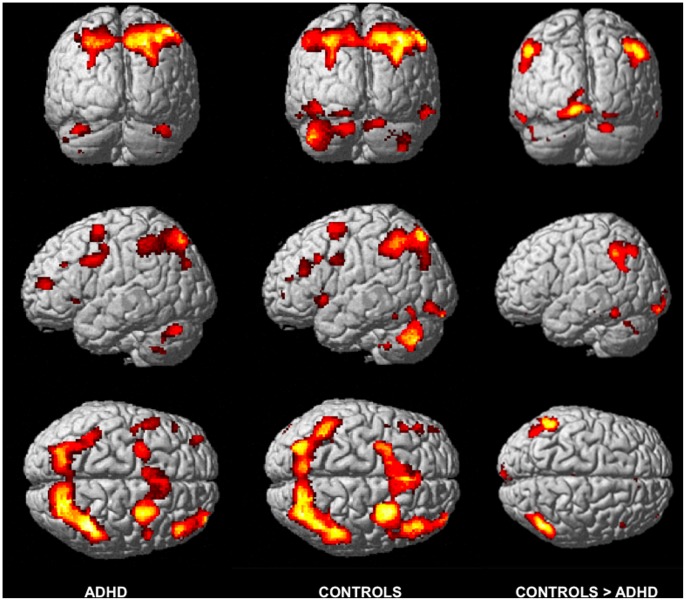
Working memory-related common and specific neural activity patterns in ADHD. Left column: WM-related activation (N2> N0) in ADHD children. Middle column: WM-related activation (N2> N0) in Control children. Right column: higher WM activation (N2> N0) in Control than in ADHD children (interaction effect). All effects are displayed at p^unc^ <0.001, superimposed on the ICBM standardized anatomical template.

**Figure 2:**
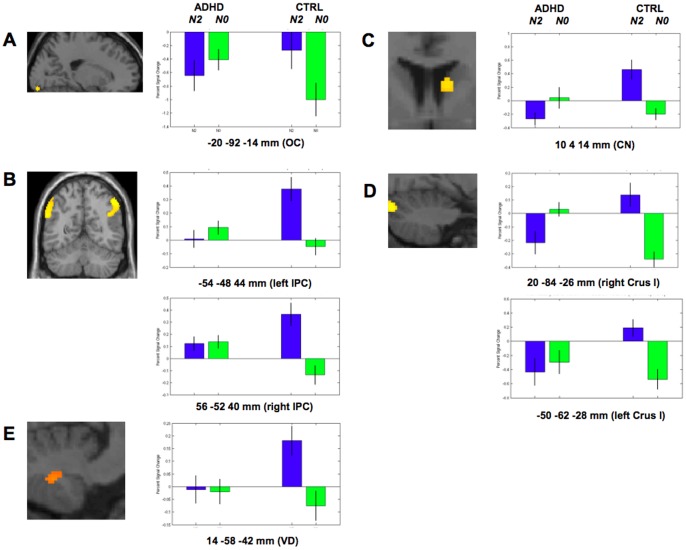
Percent BOLD signal changes from baseline levels in N2 and N0 conditions in Control and ADHD children. OC: occipital cortex; IPC: inferior parietal cortex; VD: ventral dentate (cerebellum); CN: caudate nucleus; Crus I (cerebellum). Activated areas are displayed at p^unc^ <0.001, superimposed on the ICBM standardized anatomical template.

In line with previous findings in healthy adults [9], a conjunction analysis [26] revealed higher cerebral activity in ADHD and Control participants in the N2 than in the N0 condition in a distributed network (Figure 1) mainly encompassing bilateral fronto-parietal areas and the cerebellum (Table 2). Also in line with prior publications showing higher WM-related activity in Control than ADHD participants, our analyses disclosed interaction effects between task condition (N2 vs. N0) and group (Control vs. ADHD) factors bilaterally in the inferior parietal lobule [15], [17], the right cerebellar lobule (VIIa) [16] and the caudate nucleus [15] (*ps* ^svc^ <.05), and in the left calcarine gyrus [12] (*p* ^corr^ <.05; Figure 1c and Table 3). Interaction effect was also significant in the cerebellar ventral dentate (VD; p^svc^ <.05), an output nucleus of the cerebellum projecting onto frontal cognitive areas associated with WM processes [27]. Data inspection revealed that interaction effects in the left calcarine gyrus were actually due to more decreased blood oxygen-level dependent (BOLD) responses in the N2 than in the N0 condition in ADHD children, whereas deactivation was more pronounced in the N0 than N2 condition in Control children (Figure 2.a). At variance, the N2 condition elicited increased responses in the left and right inferior parietal gyri (Figure 2.b) and in the ventral dentate (Figure 2.e) in Controls, an effect that was not present in ADHD children. Likewise, caudate nucleus activity increased in the N2 condition in Controls but decreased below baseline levels in the same condition in ADHD children (Figure 2.c). Finally in the cerebellum, activity was markedly decreased in the N2 condition in ADHD children, at variance with Controls presenting a marked deactivation in the N0 condition. Parallel deactivation in the N0 condition was also found in the left cerebellum in ADHD (Figure 2.d).

**Table 2:** WM-related activations (N2> N0) in ADHD and Control children.

Anatomical area	H	K	T	x y z (mm)	CJ
**ADHD group**					
Superior parietal	R	6403	12.25	46–46 60*	
- Superior occipital	R		10.46	26–72 40*	CJ*
- Inferior parietal	R		8.53	34–46 42*	CJ*
Superior frontal	R	1955	7.67	32 0 66*	
- Middle frontal	R		7.39	12 22 46*	
	R		7.34	30 4 54*	CJ*
Middle frontal	R	1000	7.05	46 36 30*	CJ
- Superior frontal	R		5.83	34 60 10	
- Middle orbital	R		4.84	42 56 −6	
Medial frontal	L	417	5.66	−22 4 48	
- Superior frontal	L		5.44	−28 0 60	
Precentral	R	203	5.02	48 6 32	CJ
	L	374	4.84	−48 6 34	CJ
	L		4.73	−42 0 34	
Cerebellar lobule VIIa,Crus I	L	314	5.69	−32 −62 −32	CJ
	R	300	8.18	38 −66 −30*	
**Control group**					
Precuneus	R	7224	8.35	16–68 46	CJ*
- Superior parietal	R		7.94	18–74 52	
- Inferior parietal	R		7.93	54–42 48	CJ*
Superior medial	–	4997	8.28	−2 24 44	
- Middle frontal	R		7.89	32 10 56	CJ*
- SMA	L		7.75	−10 14 52	CJ
Inferior temporal	L	429	6.43	−38 −4 30	
	R	248	6.12	54 −44−14	
- Middle temporal	R		4.17	68 −38−14	
Insula	R	364	5.43	34 20 0	CJ
Thalamus	R		5.10	8 −20 18	
	L	503	6.25	−10 −1618	
- Thal.(ventr. ant. nucleus)	L		5.84	−16 −4 14	
Cerebellar lobule VIIa,Crus I	R	530	6.20	38 −62−38	
	L	1306	7.69	−38 −68 −36	CJ
	L		6.12	−48 −68 −32	CJ
	L		5.18	−14 −82 −30	

Brain areas in which BOLD response is higher in the N2 than in the N0 condition in ADHD (top) and Control (bottom) groups. Coordinates x y z (mm) in MNI standard stereotactic space. T = t-statistic value. H = Right or Left hemisphere. K = cluster extent. CJ (conjunction analysis): areas commonly activated in ADHD and Control groups. All results significant at the voxel level p<0.001 uncorrected, except * after correction for multiple comparisons in the whole brain volume (p^corr^ <.05).

**Table 3:** Higher WM-related activation in Control than ADHD children.

Anatomical area	H	K	T	x y z (mm)
Inferior parietal	L	593	5	−54 −48 44** (a)
- Angular gyrus	L		3.99	−56 −58 28
Angular gyrus	R	694	4.92	46 −64 48** (b)
- Inferior parietal	R		4.12	56 −52 40
Left inferior temporal	L	60	4.81	−60 −50 −18
Posterior cingulate	L	121	3.81	−4 −44 18
	R		3.79	2 −44 18
Middle cingulate	L	47	3.75	−6 −30 34
Calcarine gyrus	L	476	5.99	−10 −94 −12*
	**L**		3.75	−4−100 6
Lingual gyrus	R	38	3.76	20 −92 −14
Right caudate nucleus	R	24	3.69	10 4 14** (c)
Right cerebellum(lobule VIIa Crus I)	R	103	4.3	20 −84 −26** (a)
Left cerebellum(lobule VIIa Crus I)	L	29	3.78	−50 −62 −28

Brain areas in which BOLD response is higher in the N2 than in the N0 condition, and more so in Control than ADHD children. Coordinates x y z (mm) in MNI standard stereotactic space. T = t-statistic value. H = Right or Left hemisphere. K = cluster extent. All results significant at the voxel level p<0.001 uncorrected, except * after correction in the whole brain volume (p^corr^ <0.05) or ** after correction in a small ROI volume (p^svccorr^ <.05) and cluster extent = 20 voxels. Regions of interests (ROI) taken from [a] Kobel et al. 2005, [b] Vance et al. 2007 and [c] Silk et al. 2005.

### Distinctive Functional Connectivity Patterns in ADHD (see Table 4 and Figure 3)

**Figure 3:**
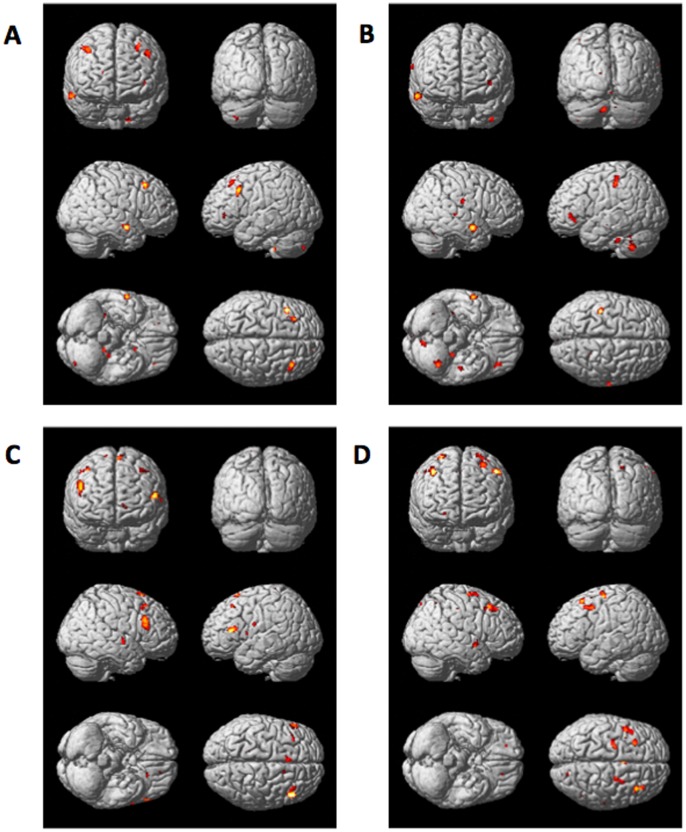
Functional connectivity patterns in ADHD. Brain areas in which activity is more tightly coupled with activity in the source area (A = left occipital, B = right cerebellum, C = inferior parietal, D = right caudate nucleus) in the working memory (N2) than the control (N0) condition, and more so in ADHD than Control children. Functionally connected regions are displayed at p^unc^ <0.001, superimposed on the ICBM standardized anatomical template.

Above specific activation/deactivation patterns in regional cerebral activity, we reasoned that ADHD children might establish distinctive functional neuroanatomical connectivity patterns during WM performance, allowing them to succeed to the task. To test this hypothesis, we computed psychophysiological interaction (PPI) [28], [29] analyses aimed at showing brain regions where activity is more tightly coupled with activity in the reference area in the N2 than the N0 condition, and more so in ADHD than Control children (Figure 3). Coordinates of interest (COI) for the 4 source areas were selected based on results from the interactions described above (see Table 3). PPI analyses revealed that activity in the left calcarine gyrus (standard coordinate −10 −94 −12 mm) was more tightly coupled during the WM (N2) than during the reference (N0) condition, and more so in ADHD than Control children, with activity in the middle frontal gyrus, the right middle and superior temporal gyri and the right fusiform gyrus, the right putamen and the left cerebellum lobule X (*ps* ^unc^ <0.001), known to participate in WM processes. The reverse contrast was non significant, i.e. we failed to disclose any area in which activity was more correlated with calcarine activity in Control than ADHD children. The same analysis conducted with the right cerebellum as COI (20 −84 −26 mm) revealed a highly significant tighter coupling in ADHD than Controls with activity in an area compatible with the brainstem red nucleus (−2 −26 −2 mm; p^corr^ <0.01). Data inspection revealed that correlation coefficients between the right cerebellum and the red nucleus area were positive but higher in ADHD than controls in the N2 condition (average *r* N2 = 0.18±0.04 vs. 0.1±0.15), and equally negative in the N0 condition (average *r* N0 = −0.07±0.17 vs. −0.09±0.16). Similar connectivity patterns with the right cerebellum were disclosed in the right amygdala, middle temporal gyrus and precuneus, left middle and frontal inferior gyri, right lingual gyrus and left cerebellum (all *ps* ^unc^ <0.001). Likewise, activity in the left inferior parietal COI (−54 −48 44 mm) was more tightly coupled in ADHD than Controls with activity in the bilateral inferior and middle frontal gyri, in the right superior temporal gyrus, in the left supplementary motor area (SMA) and anterior cingulate cortex (*ps* ^unc^ <0.001), also involved in WM processing. Finally, right caudate nucleus COI (10 4 14 mm) activity was more tightly coupled in ADHD subjects than Controls with activity in right putamen, right insula lobe, right pallidum, bilateral middle frontal gyrus and the right superior frontal gyrus (*ps* ^unc^<0.001).

**Table 4:** Psychophysiological interaction analyses.

Source area	Connected areas [N2> N0]by [ADHD > Control]	H	K	T	xyz (mm)
***Left occipital (***−***10*** −***94*** −***12 mm)***	Middle frontal gyrus	L	92	4.57	−42 16 40
		L	44	3.61	−30 26 52
		R	81	3.53	38 24 44
	Middle temporal gyrus	R	84	4.15	62 −4 −18
		R	38	3.38	30 −56 26
		R	116	4.77	60 −2 −18
	Superior temporal gyrus	R		3.73	52 −8 −12
	Fusiform gyrus	R	29	3.81	34 −36 −24
	Putamen	R	50	4.55	32 −16 0
	Cerebellum, lobule X	L	25	4.05	−18 −36 −48
***Right cerebellum (20*** −***84*** −***26 mm)***	Red nucleus	–	889	6.53	−**2** −**26** −2 *
	Amygdala	R	137	4.52	30 −6 −10
	Hippocampus	R		4.36	24 −32 −6
	Inferior frontal gyrus	L	63	3.95	−38 38 2
	Middle frontal gyrus	L	75	3.89	−30 0 40
	Lingual gyrus	R	507	4.46	12 −36 −4
		R		4.26	12 −68 2
	Precuneus	R		4.2	12 −48 16
	Postcentral gyrus	L	103	4.06	−40 −30 48
	Cerebellum (VIIa, CrusII)	L	130	4.09	−38 −54 −44
	Cerebellum (VIIa, CrusI)	L		3.6	−34 −52 −34
***Left inferior parietal (***−***54*** −***48 44 mm)***	Inferior frontal gyrus	R	192	4.28	48 22 30
		L	124	3.98	−54 28 14
	Middle frontal gyrus	R	58	3.91	40 26 52
		L	21	3.58	−32 26 50
	Superior temporal gyrus	R	30	3.94	54 −8 −4
		L	21	3.52	−42 24 48
	SMA	–	46	3.87	−2 20 66
	Anterior cingulate	L	38	3.68	−14 48 −2
***Right caudate nucleus (10 4 14 mm)***	Middle frontal gyrus	L	55	4.01	−30 22 58
		L	92	3.87	−48 6 48
		R	117	3.87	38 22 48
	Superior frontal gyrus	R	57	3.74	26 −8 68
		R		3.69	30 2 68
	Putamen	R	358	4.52	30 −8 4
	Insula	R		4.31	42 2 −4

Brain areas where coupling with the source area (coordinate of interest [COI]) is higher in the N2 than in the N0 condition, and more so in ADHD than Control children. Coordinates x y z (mm) in MNI standard stereotactic space. T = t-statistic value. H = Right or Left hemisphere. K = cluster extent. All results significant at the voxel level p<0.001 uncorrected, except * after correction in the whole brain volume (p^corr^ <0.05) and cluster extent >20 voxels.

## Discussion

In the present fMRI study, we investigated changes in regional cerebral activity and functional connectivity within brain networks underlying WM processes in children with ADHD. Importantly, our ADHD children population was carefully selected, naïve for any medication and devoid of the often-present co-morbidity, and behavioural performance was at the same level than in Controls, thus discarding the hypothesis that brain activity differences could be due to these confounding parameters. Possible limitations in the interpretation of our results are linked to the facts that having observing equal performance in the WM updating condition does not automatically imply that behavioural differences could not have been observed in more challenging conditions, and that cognitive resources needed to succeed to the task may already be differentially challenged in the two populations. Also, even if usually more robust, block-design fMRI approaches make that averaged cerebral activity over a block encompasses both the component of interest (i.e. the updating process in WM) and other less controlled cognitive processes differentiating performance on a target updating task (N-back 2) and on a control detection task (N0).

Notwithstanding, our results indicate at first glance similar patterns of working memory (WM)-related cerebral activity in ADHD and Control children, also in line with previous findings in healthy adults [9]. Indeed, WM-related responses were found in a large cerebral network encompassing the bilateral premotor and dorsal cingulate/medial premotor cortex including the supplementary motor area (SMA), the bilateral dorsolateral and ventrolateral prefrontal cortex, the frontal pole, and bilaterally the medial posterior parietal cortex including the precuneus, the inferior parietal lobes, the medial/lateral cerebellum and the thalamus. Notwithstanding, between-group comparisons revealed decreased activation patterns in ADHD children in a widespread cortico-subcortical network encompassing the bilateral occipital and inferior parietal lobes, the caudate nucleus, the cerebellum and the functionally connected brainstem nuclei. As our study was performed in an homogeneous group of children with ADHD, in whom behavioural performance was unimpaired, these results yield evidence for different distributed networks in WM-related processes relevant to ADHD.

Decreased WM-related activity in the left occipital region is partially reminiscent of previous findings in ADHD adults [12], also in line with the report of a negative correlation between right inferior occipital activity and scores of inattention at the Conner’s scale, supporting an additional link between occipital activity and WM and/or visual attentional strategies [18]. We additionally evidence here for the first time distinctive functional connectivity patterns in ADHD between occipital and cerebellar, striatal and neocortical regions involved in WM. Decreased activation has been observed during information maintenance in WM in the same occipital region, a phenomenon thought to participate in visual processing and top-down attentional modulation of posterior cortical activity [30]. In this respect, specific deficits in top-down attentional control have been reported in children with ADHD, in association with a functional disconnection between frontal and occipital cortices [31]. Hence WM-related deactivations in the occipital cortex may be linked to an organized mode of brain function, suspended as a necessary process to favour or optimize other brain resources necessary to perform on more complex components of the ongoing task [22].

Inferior parietal cortex (IPC) activity remained close to baseline under WM condition in ADHD children, whereas it markedly increased in Controls. A lack of WM-related activation in the IPC in ADHD corroborates findings from prior fMRI studies conducted in children, adolescent and adult populations with ADHD [15], [16], [32]. At variance, we did not replicate findings of decreased superior parietal cortex activation in ADHD children [16] and adolescents [15]. Notwithstanding, our and past results consistently highlight a dysfunction in ADHD in parietal areas recognized to play an important role in attention and spatial processing. Following a meta-analysis of normative fMRI studies, the IPC but not the superior parietal lobe (SPL) is a major activation cluster in fMRI studies using various versions of the N-back WM paradigm [9], which may explain an absence of effect in the SPL.

WM-related changes in striatum activity have been previously reported in the ADHD [15], [17]. In the present study, whereas activity in the caudate nucleus, a region highly innervated by dopamine projections, increased in controls in the WM N2 condition, it decreased below baseline level in the ADHD population. The caudate nucleus is a crucial component in neural networks involved in executive and cognitive control of attention and WM, playing a pivotal role in the relay of connections between the frontal cortex and striatum [19]. Furthermore, levels of caudate activation are related to specific processes underlying different aspects within WM, with information manipulation associated with higher signal intensity than retrieval [33]. Dissociable striatal contributions to ADHD have also been highlighted [34], suggesting that executive function deficits are linked to anterior striatal activity [19]. Interestingly, adolescents with ADHD both improved task performance and demonstrated decreased functional connectivity between middle frontal gyrus (MFG) and striatal regions compared to off medication on WM tasks [35]. It suggests that there is an increased demand on the frontal circuitry in non-medicated ADHD subjects, supporting the hypothesis that basal ganglia function could lead to compensatory increase in activation in the prefrontal cortex in subjects with ADHD [35]. Our own results demonstrating functional connectivity between caudate nucleus and MFG in ADHD children during WM are in agreement with this hypothesis. Additionally, morphometric MRI studies have found larger anatomical differences between ADHD and Controls in a set of regions including the right caudate, although between-study discrepancies make results globally inconsistent [13], [36]. Still, significant reductions in both right and left ventro-striatal volumes provide neuroanatomical evidence of alterations in the ventral striatum of ADHD children [37]. Initially smaller caudate volume also normalizes in ADHD males during late adolescence, possibly reflecting the clinical evolution since some ADHD symptoms tend to decrease with age in certain patients [38]. Also, striatal hypoperfusion with methylphenidate-related increase has been reported in ADHD in SPECT and PET imaging studies and functional neuroimaging studies have corroborated the important role played by the striatum in cognitive inhibitory deficits by showing reduced activations in frontal and striatum regions [39]. Altogether, the striatum and its connectivity continue to represent a prime target for future imaging studies in ADHD.

Cerebellar activity at rest in adult ADHD participants was previously found increased in the vermis after methylphenidate administration normalizing behavioural symptoms, and associated with ADHD ratings in Crus II [40]. Under WM conditions, decreased activity in right cerebellum Crus I in ADHD children was observed in ADHD children of the same age range than in the present study [16], as well as in the posterior cerebellum in ADHD adults [41]. Differences in Crus I activity between ADHD and Control children provides additional evidence for genuine functional abnormalities of the cerebellum in ADHD. Taken together, there is now robust and growing evidence for a role of the cerebellum that expands beyond motor control, with replicable cerebellar responses in a variety of domains including language, attention, executive functions, spatial processing and verbal WM that affects cognitive processing [42]. Additionally, specific neocerebellar regions are involved in distinct cognitive functions that participate in the executive control networks. Especially, lobules VI and VII (Crus I and Crus II, respectively) may selectively contribute to the parallel cortico-cerebellar loops involved in executive control and WM [43]. A broad functional lateralization of the cerebellum has also been demonstrated, with a preferential involvement of the right and left cerebellum in verbal and spatial processes, respectively [44]. Structural cerebellar abnormalities have been documented in ADHD involving among other subregions the lobule VII, as well as overall volume reductions in the right cerebellum [39]. A longitudinal case-control study [45] using volumetric regional measure further reported that whereas ADHD subjects exhibit a non-progressive volume decrease in the superior cerebellar vermis (including Crus I/lobule VIIA), those patients with worse clinical outcome additionally exhibit progressive volume reductions in the inferior posterior cerebellar lobes. It suggests that non-progressive deficit in the superior vermis in ADHD patients may represent a neuroanatomical basis for fundamental deficits in cognitive and affective processing that are resistant to plastic developmental changes in ADHD [45].

Additionally, our findings are the first to disclose tightened positive relationships under WM load in ADHD between cerebellar activity and BOLD signal changes in a brainstem area compatible with the red nucleus (RN). Available data suggest anatomical and functional relationships between the RN and a widespread sensorimotor, limbic and associative network that mainly plays a modulatory role in complex sensorimotor and cognitive processes such as WM [44]. In this respect, the RN could relay information in the phonological loop passing through the cerebellum for phonological WM necessary for speech. Additionally, a brain resting state study reported that the RN displays strong functional coherence with associative prefrontal, insular, temporal, and parietal cortex, supporting a cognitive role, with clusters also observed in occipital cortex [46]. The precise function played by the RN in ADHD symptoms remains to be elucidated.

Finally, our results failed to evidence any significant WM-related differences between ADHD and controls in prefrontal regions, although considered a critical neural substrate for WM in many studies [12], [15], [41]. This lack of differences may be due to a more limited involvement of prefrontal regions in WM-related processes in childhood. Indeed, studies having investigated the neural patterns associated with WM in healthy children [7], [8], [10], [11] have reported roughly similar activation patterns during WM than in adults, but also highlighted different developmental networks in children, that may reflect different cognitive strategies and functional brain organization. While activation patterns in adult predominate in frontal and parietal regions, in children most pronounced activation patterns are found in the premotor and parietal cortex, anterior insula, caudate/putamen, and the cerebellum [7]. A longitudinal study also provided evidence that, although most individuals recruit prefrontal cortex as expected during a WM task, this recruitment is correlated with behaviour only in late adolescence [46]. Consequently, marked differences in prefrontal cortex activity during WM in children are not expected. Notwithstanding, we have evidenced functional connectivity patterns between striatal and prefrontal regions, suggesting a cooperative involvement in WM-related networks in ADHD. Alternatively, one may hypothesise that variations in frontal activity are mostly related to differences in behavioural performance, and consequently have been dampened in our experimental design, ADHD and Control children being equated on behavioural performance. Supporting this hypothesis, a supplementary analysis comparing ADHD children with a performance lower (n = 8) or higher (n = 11) than the median split UP score (reflecting the updating process in working memory) disclosed higher cerebral activity in middle frontal (standard coordinates −36 6 62 mm) but also middle occipital (34–78 28 mm) and inferior parietal (64 −26 30 mm) and precentral (46 −26 34 mm) regions (all p<.001 uncorrected). Future investigations are needed to assess the contribution of these parameters as well as anatomical and temporal functional connectivity, taking into account the heterogeneous development and maturation of brain networks in the ADHD. Nowadays, our results point towards the existence of specific neuroanatomical patterns of brain activity, within functionally related networks, which may constitute the neural underpinnings of the cognitive architecture of ADHD.

## Materials and Methods

### Participants

Forty-two children aged 8–12 years and one of their parents gave their written informed consent to participate in this study approved by the Ethics Committee of the Erasme University Hospital, ULB, Bruxelles, Belgium. Out of 42 participants, 3 children with ADHD were excluded from the analyses following the discovery of anatomical brain abnormalities, 2 children with ADHD were excluded due to excessive head motion during MRI scanning, and 4 children (2 with ADHD, 2 healthy) were excluded based on insufficient performance during the N-back task (>1.96 SD below mean group performance).

Behavioural and neuroimaging analyses were thus conducted on 19 right-handed children fulfilling DSM-IV criteria for the ADHD combined type (9 boys) and 14 healthy volunteers (8 boys). Mean age was 10.75±1.31 years in ADHD and 10.05±1.28 years in control children (*t* = 1.53,df = 31, p = 0.13). Population consistency was also ensured with respect to handedness, age range, diagnosis of combined-type and absence of co-morbidity. Children with ADHD were recruited from the Department of Neuropediatrics, outpatient clinic in Erasme Hospital, ULB University. Healthy children agreed to participate upon announcement or personal query. All participants were identically assessed by the same child psychiatrist (IM). Diagnosis for ADHD was based on clinical features including typical history and behavioural reports. The Kiddie Schedule for Affective Disorders and Schizophrenia for School Aged Children-Present and Lifetime Version (K-SADS-PL [47]), was completed at screening for each subject to establish the diagnosis according to the DSM-IV criteria. Exclusion criteria in ADHD and controls were the presence of psychiatric co-morbidity, history of prematurity, current and past medical and neurological disorders and contraindications to MRI. All children were living in a family home and were attending normal primary schools, without educational problem, and had a scale IQ above 85 as measured by the age-appropriate Weschler Abbreviated Scale of Intelligence, WASI [48]. Finally, all children were naïve for any medication and had never been treated with any psychotropic drug during lifetime.

### Working Memory N-Back Task

WM performance and underlying cerebral activity were measured using a verbal N-back task under two different conditions [5], [9]. In both cases, stimuli were black numbers (Arial font, size 74) displayed on a white background on the centre of the screen, successively presented in pseudo-random order. In the vigilant/control 0-back (N0) condition, subjects had merely to press a button with the right hand whenever the number “2” was displayed. In the WM 2-back (N2) condition, subjects had to press the button when the displayed number was identical to the number displayed two trials before. During the fMRI session, subjects were administered 5 blocks in the N0 condition alternated with 5 blocks in the N2 condition. Each block consisted of a sequence of 30 trials (including 10 targets) each displayed for 1750 ms with an interstimulus interval of 250 ms. Each block was followed by a resting period of random duration ranging 11–16 seconds, during which the instruction relative to the forthcoming condition was displayed (i.e. either “number 2” [N0] or “same than two numbers before” [N2]). A fixation cross replaced the instruction 2.5 seconds before the start of a novel series of 30 numbers. All participants performed the whole task outside of the fMRI environment once before scanning. During the fMRI session, stimuli were projected on a translucent screen that was seen via a mirror fixed to the head coil and located in front of the subject, and responses were made with the right hand on a commercially available MRI compatible keypad system (fORP; Current Design, Vancouver) connected with a PC. The timing of MR image acquisitions and stimuli presentations was synchronised using the clock signal of the MRI scanner. Head stabilization was achieved using a head-restraining foam and MR scanner noise was attenuated using foam earplugs and headphones.

### fMRI Data Acquisition and Image Analysis

Data were acquired on a Philips Achieva 3-T (Philips Medical Systems, Best, the Netherlands) scanner using a T2* sensitive gradient echo (EPI) sequence (TR = 2130 ms, TE = , 40 ms, FA 90°, SENSE acceleration factor 2.5, matrix size 64×64×32; voxel size: 3.06×3.06×3 mm^3^). Thirty-two contiguous transverse slices were acquired, covering the whole brain. Anatomical images were obtained using a T1-weigthed sagittal 3D TFE sequence (TR 1960 ms, TE 4.60 ms, TI 1040 ms, flip angle 8°, FOV 250×250 mm^2^, matrix size 320×320×160, interpolated voxel size: 0.78×0.78×1.0 mm). The MR scanner was equipped with the Quasar imaging gradients (maximum amplitude and slew rate: 30 mT/m and 200 mT/m/ms) and a 8-channel SENSE head coil.

Functional MRI data were pre-processed and analyzed using Statistical Parametric Mapping SPM8 (Wellcome Department of Cognitive Neurology, London) software implemented in MATLAB 7.8 (Mathworks Inc., Sherbom, MA). The first five functional volumes in the acquisition were discarded to avoid transient spin saturation effects. Preprocessing included realignment and adjustment for movement related effects, co-registration of functional and anatomical data, spatial normalization into standard stereotactic MNI space and spatial smoothing using a Gaussian kernel of 8 mm full width at half maximum (FWHM). Subjects (n = 2) showing excessive scan-to-scan head motion (>4 mm) were excluded from the analyses. Additionally, the magnitude of head motion at each time point for translation and rotation parameters was obtained for each subject, and averaged within each group. No between-groups difference was evidenced using either the maximum head motion or the mean head motion measurements (ps >0.8), indicating similar movement patterns during scanning.

Data were analysed using a mixed-effects model aimed at showing a stereotypical effect in the population from which the subjects were drawn [49]. For each subject, a first-level intra-individual analysis aimed at modelling data to partition observed neurophysiological responses into components of interest, confounds and error, using a general linear model [50]. The regressors of interest were built using box cars positioned at each block (N2 and N0) presentation. These regressors were secondarily convolved with the canonical hemodynamic response function. Movement parameters derived from realignment of the functional volumes (translations in x, y and z directions and rotations around x, y and z axes) were included as covariates of no interest in the design matrix. High-pass filtering was implemented in the matrix design using a cut-off period of 256 seconds to remove low drift frequencies from the time series. Serial correlations were estimated with a restricted maximum likelihood (ReML) algorithm using an intrinsic first order autoregressive model during parameter estimation. Effects of interest were then tested by linear contrasts, generating statistical parametric maps [SPM(T)]. Here, the contrast of interest was the difference of activation between N2 and N0 conditions (N2 vs. N0) as the best approximation of neural activity associated with WM. Summary statistic images were then further spatially smoothed (6 mm FWHM Gaussian kernel) and entered in a second-level analysis in which subjects were treated as a random effect (RFX). One-sample *t* tests were used to assess the N2 vs. N0 contrast in the ADHD and control groups separately. Two-sample *t* tests were used for a direct comparison of the N2 vs. N0 contrast between ADHD and control subjects. Conjunction null analyses were used to identify the brain areas commonly activated in ADHD and controls in contrasts of interest [26]. Restricted maximum likelihood estimates of variance components were used to allow possible departure from the sphericity assumptions in RFX conjunction analyses [49].

Additionally, psychophysiological interaction (PPI) analyses [28], [29] were computed to test the hypothesis that areas showing group- and/or condition-specific neural activity might establish differential functional connections in ADHD than Control groups with other brain regions involved in WM. Coordinates of interest (COI) were determined based on results from RFX analyses described above. For each subject and each COI, the N2 vs. N0 contrast effect (corresponding to the summary statistic images entered in the RFX analysis) was computed at the individual level and the local maximum of activation determined in a small spherical volume in a 6 mm radius around the COI. This peak value was selected, unless identified outside of the brain structure of interest upon visual inspection of the individual normalized anatomical T1 image and verification of localization in SPM toolbox Anatomy atlas [51], in which case the maximum value that fitted the anatomical location was selected. A new linear model was then generated at the individual level, using three regressors. One regressor represented the task condition (N2 or N0). The second regressor was the average activity in a sphere (radius 4 mm) centred on the coordinate of the subject-specific peak value. The third regressor represented the interaction of interest between the first (psychological) and the second (physiological) regressors. To build this regressor, the underlying neuronal activity was first estimated by a parametric empirical Bayes formulation, combined with the psychological factor and subsequently convolved with the hemodynamic response function [29]. The design matrix also included the movement parameters. A significant psychophysiological interaction indicated a change in the regression coefficients between any reported brain area and the reference region related to the task condition. Individual summary statistic images obtained at the first level (fixed effects) analysis were then spatially smoothed (6 mm FWHM Gaussian kernel) and entered into a second-level (random effects) analysis using One-sample *t*-tests to test for condition-specific effects within each group separately, or two-sample *t*-tests for between–group comparisons. In all the analyses presented above, the resulting set of voxel values for each contrast constituted a map of the *t* statistic [SPM(T)], thresholded at p<0.001 (uncorrected for multiple comparisons). Statistical inferences were then obtained after corrections at the voxel level using Gaussian random field theory [52], either p^corr^ <.05 corrected for multiple comparisons in the whole brain volume and a minimal cluster size of 20 voxels (except for small structures), or p^svc^ <.05 corrected in a small spherical volume (radius 6–16 mm) around a priori locations of activation in structures of interest, taken from previous fMRI studies examining the N-back task in adults [12], [41], adolescents [15], and children [16], [17] with ADHD.
